# Investigation and Serologic Follow-Up of Contacts of an Early Confirmed Case-Patient with COVID-19, Washington, USA

**DOI:** 10.3201/eid2608.201423

**Published:** 2020-08

**Authors:** Victoria T. Chu, Brandi Freeman-Ponder, Scott Lindquist, Christopher Spitters, Vance Kawakami, Jonathan W. Dyal, Shauna Clark, Hollianne Bruce, Jeffrey S. Duchin, Chas DeBolt, Sara Podczervinski, Marisa D’Angeli, Kristen Pettrone, Rachael Zacks, Grace Vahey, Michelle L. Holshue, Misty Lang, Rachel M. Burke, Melissa A. Rolfes, Mariel Marlow, Claire M. Midgley, Xiaoyan Lu, Stephen Lindstrom, Aron J. Hall, Alicia M. Fry, Natalie J. Thornburg, Susan I. Gerber, Satish K. Pillai, Holly M. Biggs

**Affiliations:** Centers for Disease Control and Prevention, Atlanta, Georgia, USA (V.T. Chu, B. Freeman-Ponder, J.W. Dyal, Kristen Pettrone, R. Zacks, G. Vahey, M.L. Holshue, R.M. Burke, M.A. Rolfes, M. Marlow, C.M. Midgley, X. Lu, S. Lindstrom, A.J. Hall, A.M. Fry, N.J. Thornburg, S.I. Gerber, S.K. Pillai, H.M. Biggs);; Washington State Department of Public Health, Shoreline, Washington, USA (S. Lindquist, C. DeBolt, S. Podczervinski, M. D’Angeli, M.L. Holshue, M. Lang);; Snohomish Health District, Everett, Washington, USA (C. Spitters, H. Bruce);; Public Health—Seattle and King County, Seattle, Washington (V. Kawakami, S. Clark, J.S. Duchin);; University of Washington, Seattle (J.S. Duchin)

**Keywords:** COVID-19, SARS-CoV-2, contact tracing, serology, viruses, respiratory infections, coronavirus, 2019 novel coronavirus disease, coronavirus disease, severe acute respiratory syndrome coronavirus 2, zoonoses

## Abstract

We describe the contact investigation for an early confirmed case of coronavirus disease (COVID-19), caused by severe acute respiratory syndrome coronavirus 2 (SARS-CoV-2), in the United States. Contacts of the case-patient were identified, actively monitored for symptoms, interviewed for a detailed exposure history, and tested for SARS-CoV-2 infection by real-time reverse transcription PCR (rRT-PCR) and ELISA. Fifty contacts were identified and 38 (76%) were interviewed, of whom 11 (29%) reported unprotected face-to-face interaction with the case-patient. Thirty-seven (74%) had respiratory specimens tested by rRT-PCR, and all tested negative. Twenty-three (46%) had ELISA performed on serum samples collected ≈6 weeks after exposure, and none had detectable antibodies to SARS-CoV-2. Among contacts who were tested, no secondary transmission was identified in this investigation, despite unprotected close interactions with the infectious case-patient.

In December 2019, a viral pneumonia outbreak caused by severe acute respiratory syndrome coronavirus 2 (SARS-CoV-2) emerged in Wuhan, China, before spreading rapidly to other provinces in China and then internationally (*1*). On January 20, 2020, the Centers for Disease Control and Prevention (CDC) confirmed a US case of coronavirus disease (COVID-19), the disease caused by SARS-CoV-2, in a traveler who had recently returned to Washington state from Wuhan (*2*). We investigated contacts of the confirmed case-patient to describe transmission to inform public health recommendations and control measures.

## Methods

Washington state and local health officials interviewed the case-patient to identify contacts and activities during time of symptom onset until appropriate isolation of the patient. Because contact investigations for COVID-19 had not been conducted in the United States, the Washington State Department of Health (WA DOH), in consultation with CDC, developed contact definitions based on the best evidence available at the time, which are not necessarily consistent with those currently in use (Table 1). We tailored the contact definitions after the case-patient interview based on the known movement and activities of the case-patient. We categorized contacts into community or healthcare contacts. For this investigation, we defined community contact as any close contact (being within 6 feet of the case-patient) for a prolonged time (>10 minutes); being an office co-worker of the case-patient with close contact of any duration; contact with infectious secretions from the case-patient; or sharing a healthcare waiting room or area during the same time and up to 2 hours after the case-patient was present. Transient community interactions (e.g., grocery store cashiers) were not considered community contacts. Healthcare contact included any face-to-face interaction between healthcare personnel (HCP) and the case-patient without wearing the full personal protective equipment (PPE) that was recommended at the time of the investigation (i.e., gown, gloves, eye protection, and N95 respirator) or potential contact with the case-patient’s secretions by HCP without wearing full PPE. HCP who cared for the patient after patient isolation while wearing the full recommended PPE were monitored but not included in this contact investigation report.

**Table 1:** Contact definitions, number of contacts identified by category, and comments regarding the contact identification process during the contact investigation of an early confirmed US COVID-19 case, Washington, USA, 2020*

Type	Definition†	Contacts	Comments

Community contact	Any close contact (being within 6 feet of the case-patient) for a prolonged time (>10 min), other than office co-worker	0	None
Being an office co-worker of the case-patient with close contact of any duration	11 office co-workers	None
Contact with infectious secretions from the case-patient	0	None
Sharing a healthcare waiting room or area during the same time and up to 2 h after the case-patient was present	31 waiting room patients	7/31 of the waiting room contacts likely overlapped with the case-patient in the waiting room.
Healthcare contact	Any face-to-face interactions between an HCP and the case-patient without the HCP wearing full PPE†	6 HCP	None
	Potential contact with the case-patient’s infectious secretions by an HCP without wearing full PPE‡	2 HCP	2 HCP were EVS workers who did not have direct contact with the case-patient. They were included for potential exposure to infectious secretions from the case-patient while cleaning surfaces.

*COVID-19, coronavirus disease; EVS, environmental services; HCP, healthcare personnel; PPE, personal protective equipment. †Contact definitions were developed on January 21, 2020, before published guidance from CDC for COVID-19 was available. Contacts were identified during the time from the case-patient’s symptom onset until the case-patient was appropriately isolated. An airport shuttle driver who had contact with the case-patient before symptom onset was identified during contact tracing but was not included in this investigation. He self-monitored for 14 days and reported remaining asymptomatic. ‡At the time of the investigation, full PPE consisted of gown, gloves, eye protection, and an N95 respirator.

The local health departments actively monitored identified contacts during the 14 days after the last exposure date to the case-patient (i.e., the monitoring period). Active monitoring consisted of daily telephone calls or text messages to ask the contacts whether they had measured fever (>100.4°F or >38°C) or symptoms including cough, shortness of breath, chills, runny nose, body aches, sore throat, headache, diarrhea, nausea, or vomiting. The local health department supplied thermometers to contacts without a home thermometer. Contacts who developed signs or symptoms during the monitoring period were assessed as persons under investigation (PUIs) for SARS-CoV-2 infection, and nasopharyngeal (NP) and oropharyngeal (OP) swabs were obtained for testing.

Concurrently with mandatory active monitoring, CDC, WA DOH, and the local health departments conducted an enhanced contact investigation. This investigation was implemented after the urgent public health activities of identifying contacts and initiating active monitoring procedures were begun and was voluntary. The enhanced contact investigation included an in-depth interview using a standardized questionnaire and collection of NP and OP specimens and serum samples. We approached all identified community and healthcare contacts by telephone for participation. For contacts who agreed, we conducted the interview by telephone or during in-person household visits, depending on the contact availability at the time of the initial outreach. The questionnaire included demographic characteristics, medical history, and type and duration of the exposure to the case-patient. Exposure types assessed on the questionnaire were “physically within 6 feet of the case-patient,” “had face-to-face interaction,” “had direct physical contact,” and “traveled in the same vehicle, sitting within 6 feet of the case-patient.” For HCP, we also obtained data regarding healthcare-specific exposures and PPE use during the encounter. For all asymptomatic contacts who agreed, we collected NP and OP specimens and serum samples during the monitoring period to test for SARS-CoV-2 by molecular and serologic methods. We tested symptomatic contacts under PUI protocols. We approached participants in the initial enhanced investigation ≈6 weeks after last exposure to the case-patient for collection of follow-up serum samples.

NP and OP swabs were shipped to CDC and tested for SARS-CoV-2 using a real-time reverse transcription PCR (rRT-PCR) assay with 3 targets (N1, N2, and N3), as previously described (*3*). The human RNase P gene was used as an internal human gene to confirm RNA quality. Serum samples were tested at CDC using a SARS-CoV-2 ELISA with a recombinant SARS-CoV-2 spike protein (courtesy of Dr. Barney Graham, National Institutes of Health, Bethesda, MD, USA) as an antigen (*4*). Protein ELISA 96-well plates were coated with 0.15 μg/mL of recombinant SARS-CoV-2 spike protein and ELISA was carried out as previously described (*5*). An optimal cutoff optical density value of 0.4 was determined for >99% specificity and 96% sensitivity (B. Freeman et al., unpub. data; https://www.biorxiv.org/content/10.1101/2020.04.24.057323v1). Specimens with total SARS-CoV-2 antibody titers >400 were considered seropositive. Serum samples from the case-patient were used as a positive control and commercially available serum collected before January 2020 from an uninfected person as a negative control.

This public health investigation was determined to be non-research by CDC and WA DOH. Thus, it was not subject to review by either institutional review board.

## Results

**Figure 1:**
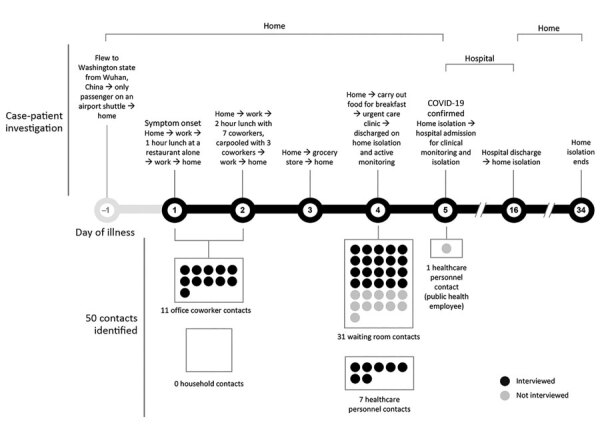
Case-patient investigation and contact identification during the investigation of an early confirmed US COVID-19 case, Washington, USA, 2020. The case-patient was asymptomatic when he arrived home from Wuhan, China. The next day, he developed a cough (day 1), followed by chills (day 2) and a subjective fever (day 3). When he arrived at the urgent care clinic (day 4), he was given a facemask and sat in the waiting room for ≈20 minutes. He was evaluated in a standard examination room, and received a chest radiograph in a radiology room down the hallway from the exam room. The case-patient was identified as meeting the Centers for Disease Control and Prevention (CDC) criteria at the time for a person under investigation for COVID-19, and specimens (nasopharyngeal and oropharyngeal swabs and serum samples) were collected for testing (*6*). He was clinically stable and discharged home pending SARS-CoV-2 test results. When COVID-19 was confirmed (day 5), the case-patient was admitted to a hospital for observation and isolation. After 11 days, he was discharged to home isolation until 2 negative sets of nasopharyngeal and oropharyngeal specimens were obtained >24 hours apart, in accordance with CDC guidance at the time (*7*). Persons exposed during transient interactions, such as restaurant waitstaff and persons encountered at the grocery store, were not considered community contacts. COVID-19, coronavirus disease.

The case-patient, a 35-year-old man, was asymptomatic when he returned home to Washington from Wuhan on January 15, 2020 (*2*). During the next 2 days, he developed a cough (day 1 of illness) and chills (day 2) while continuing to work in an office setting. He reported feeling feverish on day 3, a weekend day. On day 4, he went to an urgent care clinic, where he was identified as meeting the PUI criteria at the time for COVID-19; NP and OP swabs and serum samples were collected and sent to CDC for SARS-CoV-2 testing (*6*). Upon laboratory confirmation as a COVID-19 case-patient (day 5), he was hospitalized for isolation and clinical observation (Figure 1). 

The investigation identified 50 contacts of the case-patient while he was symptomatic, including 42 community contacts (11 office co-workers and 31 waiting room contacts at the urgent care clinic) and 8 healthcare contacts (Table 1). The case-patient lived alone, so he had no household contacts. Among the 50 contacts, the median age was 44 years (range <1–86 years); 25 (50%) were male. All 50 contacts were actively monitored daily. Eight (16%) developed symptoms, including cough (n = 6), headache (n = 5), runny nose (n = 5), sore throat (n = 4), or fever (n = 1), during their monitoring period and were assessed as PUIs; none required hospitalization (Table 2).

**Table 2:** Demographic characteristics, clinical symptoms, and laboratory testing of PUIs for COVID-19 related to an early confirmed US COVID-19 case, Washington, USA, 2020*

Characteristic	PUI, N = 8
Age, y, median (range)	41.5 (21–58)
Sex	
M	2
F	6
Contact group	
Office co-workers	2
Healthcare personnel	2
Waiting room contacts	4
Clinical symptoms	
Days from last case-patient exposure to symptom onset, median (range)	5.5 (1–11)
Cough	6
Headache	5
Runny nose	5
Sore throat	4
Body aches	3
Fever	1
Chills	1
Shortness of breath	0
Laboratory testing	
PUIs with respiratory specimens	8
PUIs with initial serum specimens	4
PUIs with follow-up serum specimens	5

*Values are no. persons except as indicated. COVID-19, coronavirus disease; PUIs, persons under investigation.

**Figure 2:**
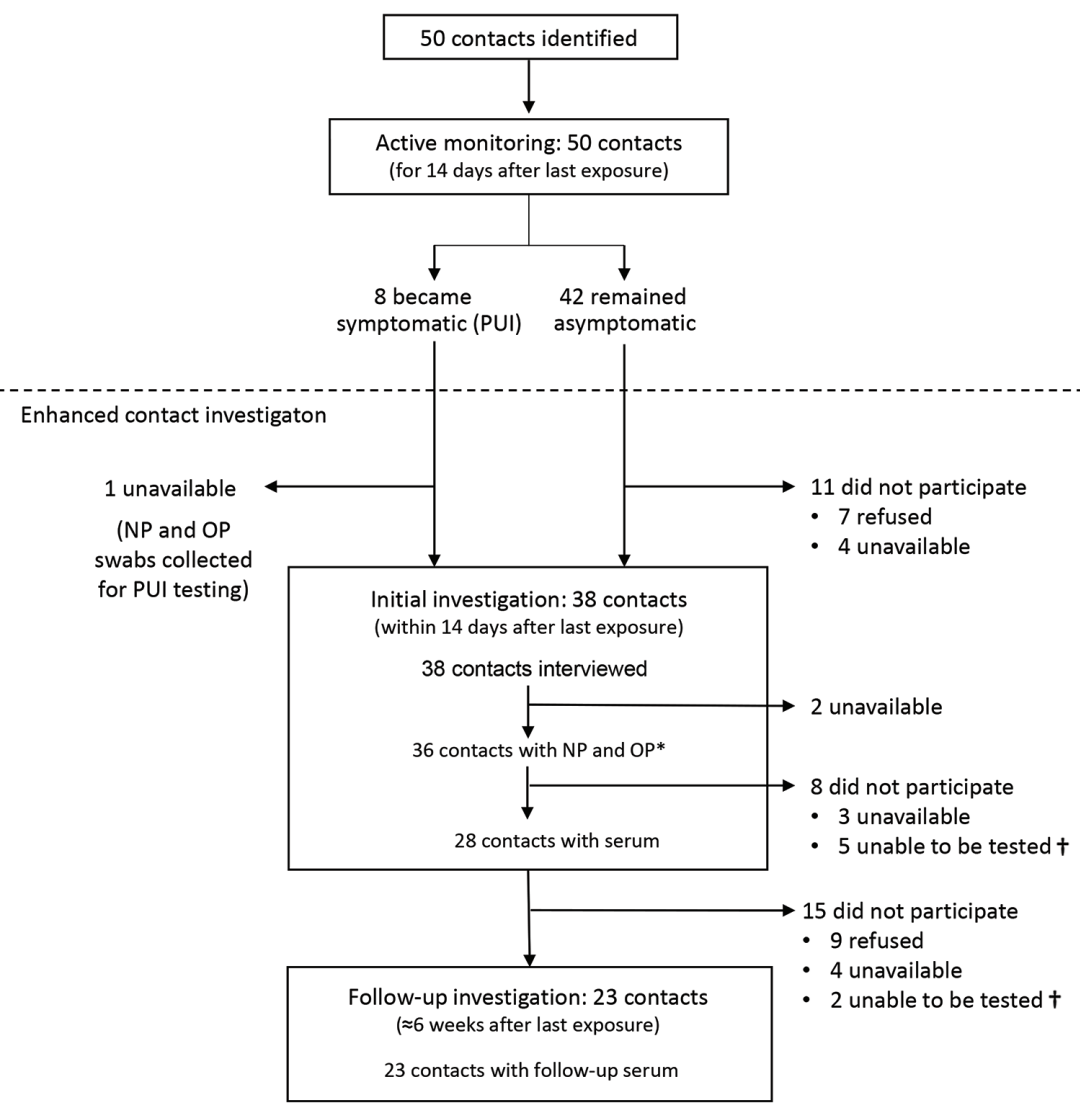
Contact investigation flowchart of identified contacts, active monitoring, and participation in the enhanced contact investigation of an early confirmed US coronavirus disease case, Washington, USA, 2020. NP, nasopharyngeal; OP, oropharyngeal; PUI, person under investigation. *Includes contacts from whom specimens obtained for PUI testing. †Specimens were unable to be tested if blood could not be obtained (n = 5) or if the standard specimen requirements for testing were not met (n = 2).

Of the 50 contacts, 38 (76%) participated in the voluntary enhanced contact investigation and completed the standardized questionnaire interview (Figure 2). Among these, 24 (63%) had >1 underlying condition, including asthma (n = 10; 26%), hypertension (n = 7; 18%), type 2 diabetes mellitus (n = 4; 11%), or pregnancy/postpartum (n = 2; 5%) (Table 3).

**Table 3:** Demographic characteristics, underlying medical conditions, exposures, and SARS-CoV-2 testing among contacts of an early confirmed US COVID-19 case participating in the enhanced contact investigation, Washington, USA, 2020*

Characteristic	All contacts, N = 38	Community contacts			Healthcare contacts
Office co-workers, n = 11	Waiting room contacts, n = 20		Healthcare personnel, n = 7†
Age, y, median (range)	45 (0–78)	39 (24–62)	53.5 (<1–78)		36 (30–56)
Sex					
M	21 (55)	9 (82)	9 (45)		3 (43)
F	17 (45)	2 (18)	11 (55)		4 (57)
Race					
White	27 (71)	2 (18)	18 (90)		7 (100)
Asian	8 (21)	8 (73)	0		0
Black	0	0	0		0
Not specified	3 (8)	1 (9)	2 (10)		0
Ethnicity					
Hispanic/Latino	5 (13)	0	3 (15)		2 (29)
Not Hispanic/Latino	28 (74)	10 (91)	14 (70)		4 (57)
Not specified	5 (13)	1 (9)	3 (15)		1 (14)
Underlying medical conditions‡					
None	15 (39)	8 (73)	3 (15)		4 (57)
Asthma	10 (26)	3 (27)	4 (20)		3 (43)
Diabetes mellitus, type 2	4 (11)	0	4 (20)		0
Hypertension	7 (18)	1 (9)	6 (30)		0
Coronary artery disease	1 (3)	0	1 (5)		0
Immunosuppressive condition or therapy	1 (3)	0	1 (5)		0
Pregnant or postpartum§	2 (5)	0	1 (5)		1 (14)
Exposure type¶					
Face-to-face interaction	16/18 (89)	11 (100)	NA		5 (71)
Direct physical contact	10/18 (56)	6 (55)	NA		4 (57)
Physically within 6 feet	16/18 (89)	11 (100)	NA		5 (71)
Within 6 feet while case-patient was coughing or sneezing	3/18 (17)	0	NA		3 (43)
Touched object handled by the case-patient	13/18 (72)	10 (91)	NA		3 (43)
In the same room	14/18 (78)	9 (82)	NA		5 (71)
Traveled in the same vehicle	3/18 (17)	3 (28)	NA		0
Active monitoring					
Days from last exposure to start of active monitoring, median (range)	4 (1–7)	6 (5–7)	4 (4–5)		1 (1–1)
Assessed as PUI during monitoring period†	7 (18)	2 (18)	4 (20)		1 (14)
Days from last exposure until symptom onset among PUIs	5 (1–11)	6 (1–11)	4.5 (3–11)		6 (6–6)
Laboratory testing					
Any testing (rRT-PCR or serology)	37 (97)	11 (100)	19 (95)		7 (100)
Enhanced contact investigation: initial specimen collection within 14 d after last exposure to case-patient					
Contacts with respiratory specimens#	36 (95)	11 (100)	19 (95)		6 (86)
Contacts with serum specimens	28 (74)	8 (73)	14 (75)		6 (86)
Days from last exposure to initial specimen collection (respiratory and serum specimens) among asymptomatic contacts, median (range)	11 (9–13)	10 (10–11)	11 (10–13)		9 (9–11)
Enhanced contact investigation: follow-up serum, ≈6 weeks after last exposure to case-patient					
Contacts with follow-up serum specimens	23 (61)	9 (82)	10 (50)		4 (57)
Days from last exposure to follow-up serum collection, median (range)	46 (44–49)	47 (46–47)	46 (44–49)		46 (45–47)

*Values are no. persons except as indicated. COVID-19, coronavirus disease; HCP, healthcare personnel; NA, not asked; PUI, person under investigation; rRT-PCR, real-time reverse transcription PCR. †One HCP PUI was not available for interview and was not included in this table. Nasopharyngeal and oropharyngeal specimens were collected for this PUI testing, and were negative by rRT-PCR for SARS-CoV-2. No serum specimens were collected. ‡Some contacts had >1 underlying medical condition. §Postpartum was defined as within 6 weeks after delivery.  ¶Exposure types were not asked for waiting room contacts, as the case-patient was anonymous to the waiting room contacts. #Included respiratory specimens obtained for PUI testing.

Eleven office co-workers were identified as having had close contact (<6 feet) with the case-patient. Four (36%) co-workers were exposed over the course of 1 day and 7 (64%) were exposed over the course of 2 days. The duration of close contact ranged from 2 to 90 minutes (<10 minutes, n = 2; 10–15 minutes, n = 3; 60–90 minutes, n = 6). All 11 co-workers reported face-to-face interaction with the case-patient, and 6 (55%) had direct physical contact (e.g., shaking hands, touching shoulder). Although the case-patient admitted symptoms of cough while at the office, no co-workers recalled being within 6 feet of the case-patient while he was coughing. On day 2 of illness, the case-patient attended a 2-hour lunch with 7 co-workers. Three co-workers traveled in the same vehicle as the symptomatic case-patient for a total of 30 minutes on the way to and from lunch.

All 8 HCP had interactions with the case-patient without wearing the full recommended PPE. One HCP was a public health employee who briefly visited the case-patient’s home and had a face-to-face conversation without wearing PPE; however, further details of this HCP’s exposure were not available because the contact did not participate in the enhanced contact investigation. The remaining 7 HCP worked at the urgent care clinic where the patient initially sought care: a receptionist, medical assistant, nurse, physician assistant, radiograph technician, and 2 environmental services (EVS) workers.

All 7 HCP at the urgent care clinic participated in the enhanced contact investigation. Of those, 5 (71%) had face-to-face interaction with the case-patient; the case-patient was wearing a facemask for most encounters. Duration of exposure among HCP ranged from 5 to 25 minutes (<10 minutes, n = 1; 10–15 minutes, n = 3; 15–30 minutes, n = 1). Three HCP had direct physical contact with the case-patient while the HCP was wearing a facemask and no gloves; HCP 1 positioned the case-patient for chest radiograph imaging, HCP 2 took the patient’s vital signs, and HCP 3 examined the case-patient and performed an OP exam. When obtaining the NP and OP swabs, HCP 3 wore an N95 respirator, face shield, and gloves but no gown. HCP 4 had direct physical contact while wearing an N95 respirator and gloves when drawing blood and processing specimens. Neither HCP wearing N95 respirators had been fit tested within the last year. The EVS workers cleaned the clinic >8 hours after the case-patient left the urgent care clinic. While cleaning, 1 EVS worker wore gloves consistently but the other did not.

The 31 waiting room contacts included patients and persons accompanying them who were likely to be in the waiting room at the same time as the case-patient or up to 2 hours after the case-patient was taken to a room. According to the sign-in sheet, ≈7 contacts overlapped in the waiting room with the case-patient. Because the case-patient was anonymous to the waiting room contacts, the 20 (65%) interviewed waiting room contacts were unable to describe their exposure type and duration. However, they consistently described being given a facemask if they had respiratory symptoms, staying >6 feet apart from other waiting room patients, and sitting <30 minutes in the waiting room. The case-patient said that he did not interact directly with any of the waiting room contacts.

Of the 50 contacts, 37 (74%) had NP and OP specimens collected and tested for SARS-CoV-2; 33 (66%) had specimens collected once, and 4 (8%) had specimens collected twice (Table 3). Of the 8 (16%) contacts who were assessed as PUIs during the investigation, all were tested for SARS-CoV-2 infection while symptomatic. Among asymptomatic contacts, the NP and OP specimens were collected a median of 11 days (range 9–13 days) from the last date of exposure. All NP and OP specimens tested negative for SARS-CoV-2 by rRT-PCR, including those from PUIs. Serum samples were obtained from 32 (64%) of the 50 contacts (Table 3). Initial serum was obtained from 28 (56%) contacts, and follow-up serum was obtained from 23 (46%) contacts; none had detectable antibodies to SARS-CoV-2.

## Discussion

This investigation identified no secondary cases among close contacts of this early US COVID-19 case-patient by molecular or serologic methods. Systematic contact tracing initiated soon after case confirmation identified office co-workers, HCP, and persons who overlapped in an urgent care clinic waiting area as contacts of the symptomatic case-patient with potential risk for infection. All 50 contacts were actively monitored daily for development of signs or symptoms consistent with COVID-19 and were assessed as PUIs if signs or symptoms developed. During the 14 days after last exposure of the identified contacts, testing of respiratory specimens by rRT-PCR of all symptomatic contacts (PUIs) and most asymptomatic contacts revealed no evidence of secondary transmission. Serum specimens collected ≈6 weeks after the last exposure for 23 (46%) contacts showed no evidence of SARS-CoV-2 antibodies, providing additional confirmation that secondary transmission did not occur among tested contacts.

The lack of transmission among contacts in this investigation is similar to findings reported in other early systematic contact investigations of case-patients with COVID-19 in January and February, in which only close household contacts were infected (*8*–*11*). One potential explanation for the lack of transmission among tested contacts may be the nature of the community exposures to the case-patient compared with the more intimate and continuous exposures that would typically be experienced by household contacts. Given the current situation of sustained community transmission of SARS-CoV-2, it is of interest that there was no evidence of transmission to co-workers, despite a 60–90 minute lunch together and travel together in a car while the case-patient was symptomatic, albeit with mild symptoms (*12*).

Similar to other reported COVID-19 case-patients, this case-patient had SARS-CoV-2 detected from NP and OP specimens at very low cycle threshold values (NP 18–19, OP 21–22) at the time of first testing at the urgent care clinic (day 4 of illness), indicating a high viral load (*2*,*13*). Of note, the case-patient wore a facemask in the urgent care clinic waiting room and during most of the healthcare encounters, except during examination and respiratory specimen collection. In addition, HCP who interacted with the case-patient all wore partial PPE, which included a facemask or an unfitted N95 respirator. Influenza studies found that patients or HCP wearing a facemask is associated with a reduced risk of nosocomial transmission (*14*). A COVID-19 contact investigation described 35 HCP who wore facemasks during a prolonged exposure to an aerosol-generating procedure for a patient with COVID-19; none acquired infection (*15*). These investigations suggest that facemasks worn by the case-patient or the HCP might have helped prevent secondary transmission of COVID-19 in the healthcare setting, although additional studies are needed.

A unique aspect of our contact investigation was the inclusion of serologic testing, which strengthened the conclusion that secondary transmission did not occur among tested contacts. Molecular testing detects viral RNA present in an active infection and is dependent on sampling location, technique, and the timing. Although the serologic assay used does not necessarily confirm current infection, it enables detection of seroconversion, indicating a history of SARS-CoV-2 infection. SARS-CoV-2 antibodies are detectable in most persons with COVID-19 within 1–3 weeks after illness onset, although more data are needed to determine whether all persons infected with SARS-CoV-2 develop detectable antibodies (*16*,*17*). Use of serologic testing complements molecular diagnostics and adds to the ability to detect asymptomatic infections or infections that occurred in persons who did not have testing performed during the acute phase of illness.

This contact investigation had limitations. The investigation involved a single case; thus, only transmissions related to specific interactions for a single case are assessed. The case-patient had no household contacts, and all HCP contacts reported using at least partial PPE. Therefore, these findings cannot be generalized for persons with other types of contacts. Furthermore, not all contacts were tested. Testing was biased toward contacts who knew the case-patient personally (office co-workers) or provided direct care for the case-patient (HCP). Most contacts were tested by rRT-PCR assay at one point during their monitoring period; we cannot exclude that timing of NP and OP swab collection could have affected the ability to capture asymptomatic infection. In addition, details of the exposure history, particularly exposure duration and frequency, are subject to recall bias. Contacts of the case-patient before symptom onset were not included in this investigation, including airplane contacts from the day before the case-patient’s symptom onset. Finally, contact identification could have been incomplete. We cannot rule out the possibility that certain interactions not captured in the contact investigation, including those classified as transient interactions, could have resulted in transmission, although unprotected prolonged exposures described in this report did not result in transmission.

This contact investigation provides detailed exposure information regarding prolonged close interactions among tested contacts that did not result in secondary transmission of SARS-CoV-2. Multiple factors likely influence transmissibility of a given COVID-19 case-patient, including viral load, symptom severity, aerosol generation, host factors in the case-patient and contact, use of protective equipment (e.g., facemask use by the case-patient), and exposure type, timing, duration, setting, and frequency. Further investigations are needed to determine host factors and exposures associated with increased transmission. 

Contact investigations coupled with laboratory testing remain crucial public health tools for identifying, isolating, and preventing additional COVID-19 cases. Serologic methods, in addition to molecular detection, are a valuable tool in improving our understanding of the rate of asymptomatic infection. Understanding more about the occurrence of asymptomatic and presymptomatic infection and its contribution to SARS-CoV-2 transmission is critical for guiding community mitigation strategies and infection prevention and control recommendations.
